# Changes in Methylation Patterns of Tumor Suppressor Genes during Extended Human Embryonic Stem Cell Cultures

**DOI:** 10.1155/2021/5575185

**Published:** 2021-09-06

**Authors:** Kyung Min Kang, Jeoung Eun Lee, Ji Eun Park, Hyunjin Kim, Hee Yeon Jang, Minyeon Go, Dong Ryul Lee, Sung Han Shim

**Affiliations:** ^1^Center for Genome Diagnostics, CHA Biotech Inc., Seoul 06135, Republic of Korea; ^2^CHA Advanced Research Institute, CHA University, Seongnam, Gyunggi-do 13488, Republic of Korea; ^3^Department of Biomedical Science, College of Life Science, CHA University, Seongnam 13488, Republic of Korea

## Abstract

While studies on embryonic stem cells have been actively conducted, little is known about the epigenetic mechanisms in human embryonic stem cells (hESCs) in extended culture systems. Here, we investigated whether CpG island (CGI) methylation patterns of 24 tumor suppressor genes could be maintained during extended hESC cultures. In total, 10 hESC lines were analyzed. For each cell line, genomic DNA was extracted from early and late passages of cell cultures. CGI methylation levels of 24 tumor suppressor genes were analyzed using methylation-specific multiplex ligation-dependent probe amplification (MS-MLPA), pyrosequencing, and real-time polymerase chain reaction (PCR). Different CGI methylation patterns of *CASP8*, *FHIT*, and *CHFR* genes were identified in between early and late passages in some hESC lines. CGI methylation levels of *CASP8* significantly increased at late passage in CHA-36, CHA-40, and CHA-42 cell lines compared to those at early passage. The CGI methylation of the *FHIT* gene was higher at late passage than at early passage in CHA-15, CHA-31, CHA-32, and iPS (FS)-1 cell lines but decreased at the late passage in CHA-20 and H1 cell lines. Different CGI methylation patterns were detected for the *CHFR* gene only in iPS (FS)-1, and the level significantly increased at late passage. Thus, our findings show that CGI methylation patterns could be altered during prolonged ESC cultures and examining these epigenetic changes is important to assess the maintenance, differentiation, and clinical usage of stem cells.

## 1. Introduction

Human embryonic stem cells (hESCs) are self-renewing, pluripotent, and undifferentiated cells derived from the inner cell mass of human blastocysts [1]. HESCs have the following characteristics: they can be grown infinitely *in vitro*, maintain a normal karyotype during long culture periods, and potentially differentiate into any kind of tissue [2].

Recent 10-year research has shown that ESCs may be affected by chromosomal abnormality or genetic change under extended cultures. Rebuzzini et al. performed a cytogenetic analysis of three mouse ESC lines during prolonged passaging *in vitro* and reported karyotype changes in all three cell lines [3]. Draper et al. detected a gain of chromosome 17q and 12 in three different hESCs [4]. Upon repeated growth in extended cultures, hESCs can rapidly proliferate by leaky cell cycle checkpoint [5–7]. This phenomenon is called “culture adaptation” which may increase growth rate, change their euploid karyotype, and make them immune to apoptosis [4, 5, 8, 9]. This process is highly similar to tumorigenesis [9]. Similarly, during prolonged cultures, human ESCs tend to lose their inherent characteristics and acquire a cancer-like phenotype [5, 10, 11].

Epigenetic aberrations can alter the function of certain genes related to tumorigenesis [12–14] and are thought to be one of the important factors that induce tumorigenesis in hESCs. Epigenetics has been defined more broadly as the dynamic regulation of gene expression by a sequence-independent mechanism, including changes in DNA methylation and histone modifications. Promoter DNA methylation of CpG islands (CGIs) is a critical epigenetic mechanism that plays a major role in switching the activity of specific genes. CGIs are highly active during development, while most CpG sites are usually methylated [14, 15]. Aberrant methylation of CGIs has been shown to be associated with the transcriptional inactivation of tumor suppressor genes [16]. Hypermethylation of tumor suppressor genes is commonly related to their inactivation and the subsequent development of cancer [12, 17, 18].

Many methods, such as methylation-specific polymerase chain reaction (MSP), methylation-specific multiplex ligation-dependent probe amplification (MS-MLPA), and methylation-sensitive high resolution melting (MS-HRM), have been widely used for the detection of methylation tendency. Among these, MS-MLPA is a fast, inexpensive, and reliable system for epigenetic characterization of tumor suppressor genes [19, 20]. Therefore, in this study, we used MS-MLPA and other methods to examine the methylation changes in CGIs at the promoter region of 24 tumor suppressor genes during extended hESC cultures.

## 2. Materials and Methods

### 2.1. Human Pluripotent Stem Cell Culture

For this study, nine hESC lines (CHA-hES 15, 20, 31, 32, 36, 40, 42, B3, and H1; WiCell, Madison, WI, USA) and one hiPSC line (iPS (FS)-1; WiCell, Madison, WI, USA) were used. All hESC lines except H1 were established and characterized in CHA Gangnam Medical Center, CHA University, Korea, after receiving approval from the Institutional Review Board (IRB) of CHA Gangnam Medical Center as previously described [21], and then registered in the National Stem Cell Registry in Korea (Supplementary Table 1). Human ESCs were cultured as previously described [21]. Depending on the ESC derivation condition, CHA-hES 15, 31, 32, 36, B3, and H1 were cultured on mouse embryonic fibroblast feeder cells with 0.1% gelatin coating (Sigma-Aldrich, St. Louis, USA) in ES-SR medium (Dulbecco's modified Eagle's medium (DMEM)/F12 supplemented with 20% Knockout Serum Replacement (SR), 0.1 mM *β*-mercaptoethanol, 1% nonessential amino acids, 100 units/mL penicillin, 100 *μ*g/mL streptomycin, and 4 ng/mL basic fibroblast growth factor (bFGF); all products from Thermo Fisher Scientific) at 37°C in a humidified 5% CO_2_ incubator. CHA-hES 20 cells were cultured on human foreskin fibroblast feeder cells with 0.1% gelatin coating in ES-SR medium. CHA-hES 40 and 42 were cultured on human endometrial fibroblast feeder cells with CELLstart (Life Technologies, New York, USA) coating in ES-SR XenoFree medium, which was the same as ES-SR medium except that KnockOut SR XenoFree (Life Technologies, New York, USA) was used instead of SR. iPS (FS)-1 was cultured in mTeSR1 medium (STEMCELL Technologies, Vancouver, Canada) according to the manufacturer's instructions. Each hPSC line was collected at early passages and late passages for this study.

### 2.2. DNA and RNA Extraction

Genomic DNA was extracted from cultured stem cells using the QuickGene DNA tissue kit (Kurabo, Osaka, Japan) according to the manufacturer's instructions. Total RNA was extracted from cultured stem cells using the RNeasy® Mini kit (Qiagen, Hilden, Germany) according to the manufacturer's instructions. DNA or RNA yield was quantified from a 1 *μ*L sample using a NanoDrop™ spectrophotometer (Thermo Scientific, Maryland, USA). Extracted DNA and RNA were stored at -80°C until further analysis.

### 2.3. MS-MLPA

DNA methylation status was analyzed by MS-MLPA using the SALSA MS-MLPA ME001-C1 tumor suppressor kit (MRC-Holland, Amsterdam, the Netherlands). This probe mix contains 26 probes (Supplementary Data 2) that detect the methylation status of promoter regions of 24 tumor suppressor genes and 15 control probes that are not affected by methylation-sensitive HhaI restriction enzyme. In total, 100 ng DNA was hybridized with 26 probes for 16 h at 60°C. The sample was divided into two parts: one was ligated with HhaI, while the other was ligated without HhaI. This digestion only acts on unmethylated sequences. PCR was performed on these samples (35 cycles of denaturation at 95°C for 30 s, annealing at 60°C for 30 s, extension at 72°C for 1 min, and final extension at 72°C for 20 min). The PCR product was combined with 9 *μ*L of Hi-Di™ formamide (Life Technologies, CA, USA) and 0.3 *μ*L of GeneScan™-500LIZ™ Size Standard (Applied Biosystems, CA, USA) and analyzed using a 3130XL Genetic Analyzer (Applied Biosystems, CA, USA). Quantification was performed using the GeneMapper software (Applied Biosystems, CA, USA).

### 2.4. Sodium Bisulfite Modification

Genomic DNA was modified using the EZ DNA Methylation-Lighting™ kit (Zymo Research, CA, USA) according to the manufacturer's instructions. The samples were subjected to the following steps in a thermal cycler (MJ Research Inc., Watertown, MA): 8 min at 98°C, 60 min at 54°C, and 4°C for up to 20 h. The DNA was purified and added to a Zymo-Spin IC™ column containing the M-binding buffer and mixed by inverting the column several times. The column was centrifuged at full speed for 30 s, and the flow-through was discarded. The column was washed by adding 200 *μ*L of M-wash buffer and centrifuged at full speed, followed by treatment with 200 *μ*L M-desulphonation buffer at room temperature (20-30°C) for 15-20 min. After incubation, the column was centrifuged at full speed for 30 s, washed by adding 200 *μ*L of M-wash buffer, and centrifuged at full speed (this step was repeated). The converted gDNA was eluted by adding 20 *μ*L of M-elution buffer to the column and spin. DNA samples were stored at -20°C until further use.

### 2.5. Pyrosequencing Analysis

We used the bisulfite pyrosequencing method for methylation analyses of *CASP8*, *FHIT*, *CDKN2B*, and *CHFR* genes. Each primer was designed using Pyrosequencing Assay Design Software v2.0 (Qiagen, Germany). The primer sequence is listed in Supplementary Data 3. The amplification was carried out according to the general guidelines suggested by pyrosequencing as follows: denaturing at 95°C for 10 min, followed by 45 cycles at 95°C for 30 s, at ∗ °C for 30 s, at 72°C for 30 s, and a final extension at 72°C for 5 min (^∗^CASP8 and FHIT: 54°C; CDKN2B and CHFR: 50°C). PCR products were confirmed by electrophoresis on a 2% agarose gel and visualized by ethidium bromide staining.

The ssDNA template was prepared from a biotinylated PCR product using streptavidin Sepharose® HP beads (Amersham Biosciences, Sweden) following the PSQ 96 sample preparation guide using multichannel pipettes. Fifteen picomoles of the respective sequencing primers were added for analysis. Sequencing was performed on a PyroMark ID system with the Pyro Gold reagent kit (Qiagen, Germany) according to the manufacturer's instructions. The methylation percentage was calculated as the average degree of methylation at CpG sites formulated by pyrosequencing.

### 2.6. Real-Time PCR

After DNase I treatment, 0.5 *μ*g of total RNA was reverse transcribed using SuperScript® III First-Strand (Invitrogen, Life Technologies™, CA, USA) according to the manufacturer's instructions. Amplified cDNAs were 1/50 diluted and mixed with BIO-RAD iQ™ SYBR® GREEN Supermix (BIO-RAD Laboratories, Singapore). The primer sequence is listed in Supplementary Data 4.

Real-time PCR was performed on these samples under the following conditions: 15 min at 95°C, followed by 40 cycles of 15 s at 95°C, 15 s at 60°C, and 20 s at 72°C on iQ™5 Optical System Software version 2.0 (BIO-RAD Laboratories, CA, USA).

Relative gene expression levels were calculated using the *ΔΔ*Ct method and normalized to 28S rRNA housekeeping gene expression.

## 3. Result

### 3.1. Methylation Analysis in the CGIs of Tumor Suppressor Genes Using MS-MLPA

All samples were examined for chromosomal copy number using MLPA 070 and 036 probe sets which were designed to detect deletion or duplication of subtelomeric and centromeric regions, respectively. The CGI methylation status was determined using MS-MLPA ME001, comprising 24 tumor suppressor genes and 15 reference probes. These 24 genes are known to be methylated in tumors but are usually unmethylated in the blood-derived DNA of healthy individuals. The promoter is considered methylated at a dosage ratio over 0.25 (25%) [16, 20]. We used normal tissue cells as a negative control and cancer cells as a positive control (Supplementary Data 5). No methylation pattern was detected in normal cells (Supplementary Data 5B). In contrast, *APC*, *DAPK1*, *IGSF4*, *RARB*, and *TIMP3* genes were found to be methylated in cancer cells (Supplementary Data 5D).

**Figure 1:**
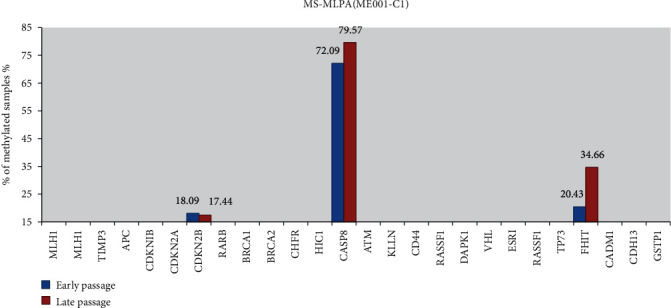
Mean value of methylation frequencies of the 25 analyzed genes in early passage and late passage of 10 cell lines (methylation cut-off value 25%).

**Figure 2:**
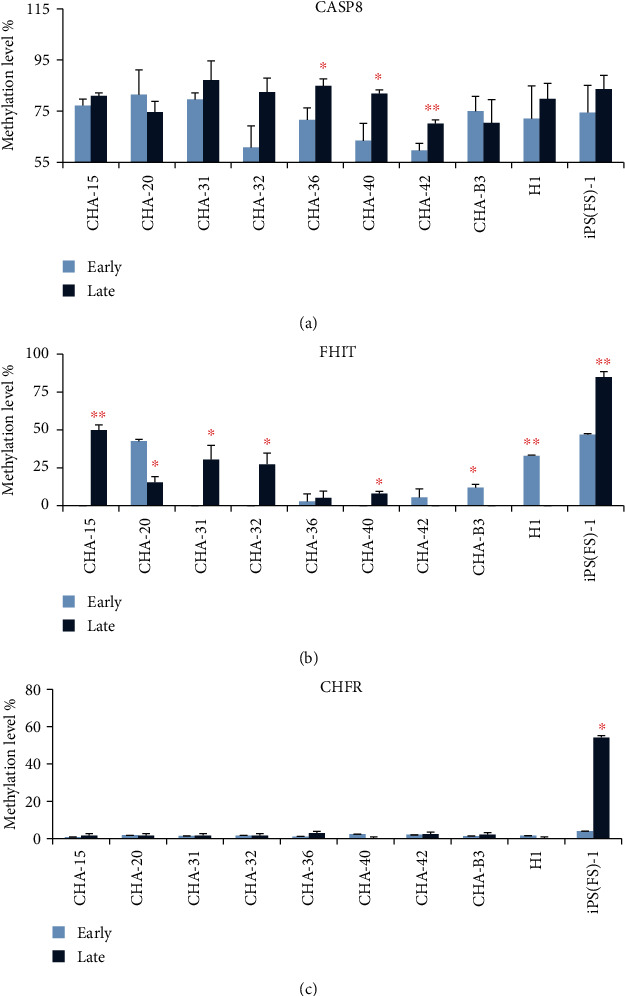
Methylation level of the three genes between early passage and late passage of 10 cell lines using MS-MLPA. Methylation level % of the *CASP8* gene (a), *FHIT* gene (b), and *CHFR* gene (c).

We performed MS-MLPA analysis on eight human ES cell lines that were established and characterized in CHA Gangnam Medical Center, CHA University, Korea, one H1 hES cell line provided by WiCell, and one iPS cell line (iPS (FS)-1, WiCell). The CGI methylation of *CASP8* and *FHIT* genes was detected in a total of 10 cell lines, while that of the *CHFR* gene was detected at late passage in iPS (FS)-1 cell line (Figure 1). The CGI methylation level of the *CASP8* gene significantly increased between early and late passages in CHA-36 (71.57: 84.90%, *p* = 0.015), CHA-40 (63.43: 81.87%, *p* = 0.029), and CHA-42 (59.70: 70.13%, *p* = 0.006) cell lines (Figure 2(a)), while that of the CGI *FHIT* gene significantly increased between early and late passages in CHA-15 (0.00: 49.90%, *p* = 0.002), CHA-31 (0.00: 30.43%, *p* = 0.030), CHA-32 (0.00: 27.37%, *p* = 0.023), and iPS (FS)-1 (46.97: 84.87%, *p* = 0.004) cell lines and significantly decreased between early and late passages in CHA-20 (42.63: 15.37%, *p* = 0.011) and H1 (32.97: 0.00%, *p* = 0.00004) (Figure 2(b)). CGI methylation level of the *CHFR* gene was detected only in iPS (FS)-1, and the level was significantly increased at the late passage (0.00: 52.87%, *p* = 0.009) (Figure 2(c)).

Additionally, MS-MLPA experiments were performed by randomly taking middle passages between early and late passages in 10 previously tested cell lines and H9 hES. The middle passage cell lines were cultured under the same conditions as those used in the previous experiments, and these were randomly selected. As a result, changes in methylation levels occur gradually during extended cell culture (Supplementary Data 6).

### 3.2. Validation of MS-MLPA Results by Pyrosequencing

We performed bisulfite-pyrosequencing analysis for *CASP8*, *FHIT*, and *CHFR* genes selected from the MS-MLPA data. Each primer, *CASP8*-126~-63 (3 CpG sites), *FHIT* 528~575 (5 CpG sites), and *CHFR* 442~478 (3 CpG sites), was designed using the Pyrosequencing Assay Design Software v2.0. The methylation percentage was calculated by the average of the degree of methylation at CpG sites.

**Figure 3:**
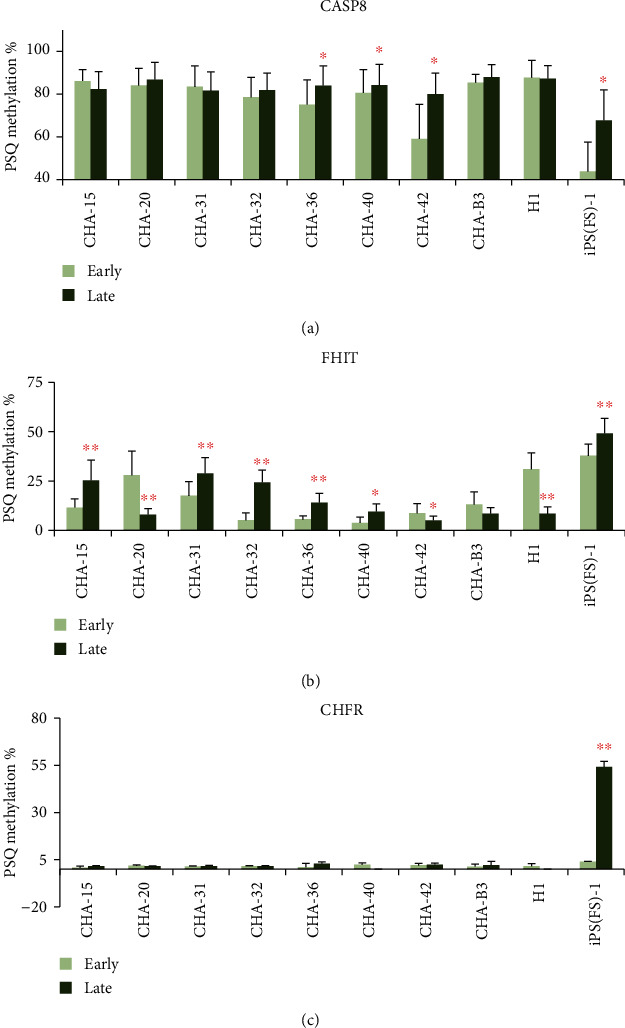
Methylation level of the three genes between early passage and late passage of 10 cell lines using pyrosequencing. PSQ methylation % of the *CASP8* gene (a), *FHIT* gene (b), and *CHFR* gene (c).

As a result, the methylation level of *CASP8* was significantly higher at the late passage than at the early passage in CHA-36 (75.18: 84.01%, *p* = 0.025), CHA-40 (80.58: 84.24%, *p* = 0.049), CHA-42 (59.03: 80.01%, *p* = 0.029), and iPS (FS)-1 (43.78: 67.75%, *p* = 0.018) cell lines (Figure 3(a)). The methylation of *FHIT* significantly increased at the late passage in CHA-15 (11.59: 25.36%, *p* = 0.007), CHA-31 (17.55: 28.88%, *p* = 0.00001), CHA-32 (5.22: 24.28%, *p* = 0.0003), CHA-36 (5.65: 14.11%, *p* = 0.005), CHA-40 (3.86: 9.46%, *p* = 0.015), and iPS (FS)-1 cell lines (37.79: 49.15%, *p* = 0.001) and decreased at the late passage in CHA-20 (27.99: 7.97%, *p* = 0.010), CHA-42 (8.79: 5.12%, *p* = 0.04), and H1 cell lines (31.06: 8.43%, *p* = 0.001) (Figure 3(b)). The methylation level of the *CHFR* gene significantly increased only in iPS (FS)-1 cell line (3.99: 54.17%, *p* = 0.001) (Figure 3(c)), consistent with the MS-MLPA data.

### 3.3. Validation of MS-MLPA Results by Real-Time PCR

**Figure 4:**
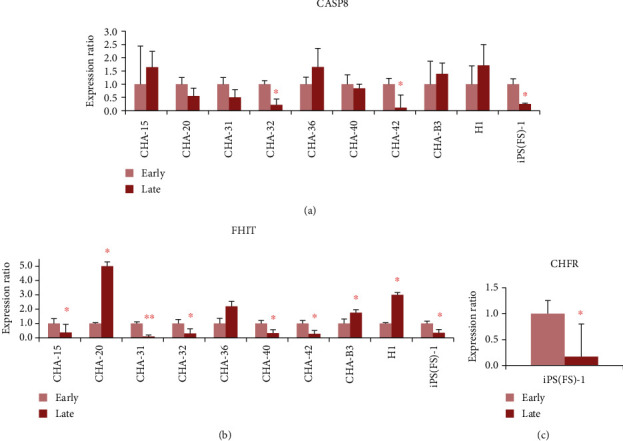
Expression level of the three genes between early passage and late passage of 10 cell lines using real-time PCR analysis. Expression ratio of the *CASP8* gene (a), *FHIT* gene (b), and *CHFR* gene in iPS (FS)-1 cell (c).

We also performed real-time PCR to confirm whether the methylation at the level of the promoter regions of the genes could affect their expression. The expression level of *CASP8* was significantly downregulated at late passage in CHA-42 (1.00: 0.11, *p* = 0.015) and iPS (FS)-1 (1.00: 0.25, *p* = 0.022) compared to their early passage (Figure 4(a)). The expression level of the *FHIT* gene was changed in 9 cell lines. It was significantly decreased at late passage in CHA-15 (1.00: 0.37, *p* = 0.023), CHA-31 (1.00: 0.09, *p* = 0.005), CHA-32 (1.00: 0.30, *p* = 0.044), CHA-40 (1.00: 0.21, *p* = 0.012), CHA-42 (1.00: 0.21, *p* = 0.021), and iPS (FS)-1 (1.00: 0.35, *p* = 0.012) cell lines as compared to that in early passage. *FHIT* expression significantly increased at passage in CHA-20 (1.00: 5.21, *p* = 0.030) and H1 (1.00: 3.00, *p* = 0.20) (Figure 4(b)). The expression level of the *CHFR* gene significantly decreased at late passage in iPS (FS)-1 cell lines (1.00: 0.17, *p* = 0.020) (Figure 4(c)).

## 4. Discussion

In this study, we attempted to find the methylation changes in the promoter regions of tumor suppressor genes in human ESCs under an extended culture system using the MS-MLPA method. We detected the methylation of *CASP8*, *FHIT*, and *CHFR* genes and found that the level of methylation changed between early and late passages in some cell lines. We identified that the changes in CGI methylation levels in these genes led to variations in their expression. Except for the methylation of *CASP8* and *FHIT* genes in CHA-36 cells, we reported consistency between MS-MLPA, pyrosequencing, and real-time PCR data (Table 1). We confirmed the change in the CGI promoter of *CASP8* or *FHIT* in nine cell lines from a total of 10 cell lines. In most cell lines (7 of 9), the CGI methylation level of *CASP8* or *FHIT* gene increased at late passage. On the other hand, the level of *FHIT* in CHA-20 and H1 cell lines decreased at late passage. The results of these two cell lines differed from those of the other cell lines, probably owing to different conditions, such as the type of cell line, feeder cell, or media. According to a recent study by Thompson et al., it was revealed that there is a difference in the methylation change depending on the culture conditions of stem cells [22]. We conducted MS-MLPA experiments by dividing each stem cell line into early, middle, and late passages to determine the effect of time in culture, and it was confirmed that methylation values gradually increased or decreased. In Garitaonandia et al.'s study, they showed that DNA methylation of hPSCs was significantly altered during extended culture under all culture conditions [23]. Based on these data, there may be differences in degree depending on the cell line or media conditions, but it was found that a gradual change of gene methylation occurred in the case of continuous subculture.

Unlike hESCs, the CGI methylation of *CHFR* was detected in iPS cell line. Previous studies comparing hiPSCs with hESCs have revealed both similarities and differences with regard to the transcriptome, genomic stability, histone modification, and DNA methylation [24–26]. Despite the limited sample size, we were able to show differences between hESCs and hiPSCs.

The gene *CASP8* encodes a member of the cysteine-aspartic acid protease (caspase) family. Sequential activation of caspases plays a central role in the execution phase of cell apoptosis. This protein is involved in programmed cell death induced by Fas and various apoptotic stimuli [27, 28]. *FHIT* plays a role in the induction of apoptosis via SRC and protein kinase B (AKT1) signaling pathways and modulates transcriptional activation by catenin beta 1 (CTNNB1), thereby contributing to the regulation of the expression of the genes essential for cell proliferation and survival, such as CCND1 and BIRC5 [29–31]. The *CHFR* gene encodes an E3 ubiquitin-protein ligase required for the maintenance of the antephase checkpoint that regulates cell cycle entry into mitosis and, therefore, may play a key role in cell cycle progression and tumorigenesis [32, 33].

CGI methylation is the main epigenetic mechanism, and changes in this mechanism play an important role in tumorigenesis. The MS-MLPA assay is a fast and sensitive method for detecting changes in the methylation of multiple genes in a single reaction. Therefore, in this study, the MS-MLPA method was applied for the simultaneous analysis of the methylation status of distinct tumor suppressor genes in human ESCs. As this study is limited to cell culture *in vitro*, further studies on knockout models are needed to assess whether methylation changes in tumor suppressor genes actually lead to the change from stem cells to tumorigenesis.

## 5. Conclusion

We showed that during extended cultures, human embryonic stem cell lines may not undergo large genomic alterations such as chromosome abnormalities but show alterations in CGI methylation levels of tumor suppressor genes. To use hESC lines for cell therapy, it would be imperative to closely verify structural variations at the chromosome or genome level as well as the epigenetic changes.

## Figures and Tables

**Table 1:** Comparison of MS-MLPA, pyrosequencing, and real-time PCR data.

No.	Cell line	Gene	MS-MLPA	PSQ	Real-time PCR
1	CHA-15	CASP8	−	−	−
		FHIT	↑	↑	↓
2	CHA-20	CASP8	−	−	−
		FHIT	↓	↓	↑
3	CHA-31	CASP8	−	−	−
		FHIT	↑	↑	↓
4	CHA-32	CASP8	−	−	↓
		FHIT	↑	↑	↓
5	CHA-36	CASP8	↑	↑	(↑)
		FHIT	(↑)	↑	(↑)
6	CHA-40	CASP8	↑	↑	(↓)
		FHIT	↑	↑	↓
7	CHA-42	CASP8	↑	↑	↓
		FHIT	−	↓	↓
8	CHA-B3	CASP8	−	−	−
		FHIT	↓	(↓)	↑
9	H1	CASP8	−	−	−
		FHIT	↓	↓	↑
10	iPS (FS)-1	CASP8	(↑)	↑	↓
		FHIT	↑	↑	↓
		CHFR	↑	↑	↓

PSQ: pyrosequencing; ↑: increased in late passage than early passage; −: no change between early and late passages; ↓: decreased in late passage than early passage; (↑): showed a tendency to increase; (↓): showed a tendency to decrease.

## Data Availability

The MS-MLPA, pyrosequencing, and real-time PCR data used to support the findings of this study are included within the article and the supplementary information file.

## Supplementary Materials

Supplementary Materials Supplemental Data 1: human stem cell lines. Supplemental Data 2: genes in the methylation-specific MS-MLPA kit (ME001-C1) tumor suppressor. Supplemental Data 3: the primers for pyrosequencing. Supplemental Data 4: the primers for real-time PCR. Supplemental Data 5: methylation frequencies (cut-off value 25%) of the 25 promoter regions in normal tissue cells and cancer tissue cells. Supplemental Data 6: (A)–(J) MS-MLPA was performed by randomly taking middle passage cell lines between early passages and late passages, and it was confirmed that their methylation values were within the trend lines of early and late passages. “Early” and “Late” under passage mean the cell line used previous experiment. In (F), (I), and (J), early passage or late passage cell lines were used as an exception because it was difficult to use middle passages.
