# Supplementary material for: Changes in Methylation Patterns of Tumor Suppressor Genes during Extended Human Embryonic Stem Cell Cultures
Source: Stem Cells Int. 2021 Sep 6;2021:5575185. doi: 10.1155/2021/5575185 (PMC8452414; doi:10.1155/2021/5575185)
Supplement: Supplementary Materials — Supplemental Data 1: human stem cell lines. Supplemental Data 2: genes in the methylation-specific MS-MLPA kit (ME001-C1) tumor suppressor. Supplemental Data 3: the primers for pyrosequencing. Supplemental Data 4: the primers for real-time PCR. Supplemental Data 5: methylation frequencies (cut-off value 25%) of the 25 promoter regions in normal tissue cells and cancer tissue cells. Supplemental Data 6: (A)–(J) MS-MLPA was performed by randomly taking middle passage cell lines between early passages and late passages, and it was confirmed that their methylation values were within the trend lines of early and late passages. “Early” and “Late” under passage mean the cell line used previous experiment. In (F), (I), and (J), early passage or late passage cell lines were used as an exception because it was difficult to use middle passages. [file 5575185.f1.docx]

|  | **Early** | **middle** | **Late** | **Feeder** | **Media** |
| --- | --- | --- | --- | --- | --- |
| **CHA-15** | p9 | p32+1  p25+19 | p75 | CF1 | ES-SR |
| **CHA-20** | p4+15 | p4+72 | p4+135 | FS | ES-SR |
| **CHA-31** | p4 | p12+45 | p12+58 | CF1 | ES-SR |
| **CHA-32** | p6 | p7+41 | p7+57 | CF1 | ES-SR |
| **CHA-36** | p5 | p60 | p98+2 | CF1 | ES-SR |
| **CHA-40** | p3 | p40 | p54 | Endo | ES-HSR |
| **CHA-42** | p4 | p37  p96 | p59 | Endo | ES-HSR |
| **CHA-B3** | p8 | p4+39 | p64 | CF1 | ES-SR |
| **H1** | p37+5 | p35+4 | p37+50 | CF1 | ES-SR |
| **H9** | p30+4 | p30+10 | P30+15 | CF1 | mTeSR1 |
| **iPS(FS)-1** | p32 | p32(17)+140 | p32+82 | F.F. | mTeSR1 |

Supplemental Data 1 Human Stem Cell Lines

| **Gene** | **Name** | **Probes** | | **Chromosomal location** | | |
| --- | --- | --- | --- | --- | --- | --- |
| MLH1 | mutL homolog 1 | 01686-L01266 | | 3p22 | | |
| MLH1 | mutL homolog 1 | 02260-L01747 | | 3p22 | | |
| TIMP3 | metallopeptidase inhibitor 3 | 02255-L03752 | | 22q12 | | |
| APC | adenomatous polyposis coli | 01905-L01968 | | 5q22 | | |
| CDKN1B | Cyclin-dependent kinase inhibitor 1B | 07949-L07730 | | 12p13 | | |
| CDKN2A | Cyclin-dependent kinase inhibitor 2A | 01524-L01744 | | 9p21 | | |
| CDKN2B | Cyclin-dependent kinase inhibitor 2B | 00607-L00591 | | 9p21 | | |
| RARB | Retinoic acid receptor, beta | 04040-L01698 | | 3p24 | | |
| BRCA1 | Breast cancer 1 | 05162-L04543 | | 17q21 | | |
| BRCA2 | Breast cancer 2 | 04042-L03755 | | 13q13 | | |
| CHFR | Checkpoint with forkhead and ring finger domains | 03813-L03753 | | 12q24 | | |
| HIC1 | hypermethylated in cancer 1 | 03804-L00949 | | 17p13 | | |
| CASP8 | caspase 8, apoptosis-related cysteine peptidase | 02761-L02210 | | 2q33 | | |
| ATM | Ataxia telangiectasia mutated | 04044-L03849 | | 11q22 | | |
| KLLN | killin, p53-regulated DNA replication inhibitor | 02203-L08261 | | 10q23 | | |
| CD44 | CD44 molecule (Indian blood group) | 03817-L01731 | | 11p13 | | |
| RASSF1 | Ras association (RalGDS/AF-6) domain family member 1 | | 02248-L01734 | | 3p21 | |
| DAPK1 | death-associated protein kinase 1 | | 01677-L01257 | | | 9q21 |
| VHL | von Hippel-Lindau tumor suppressor | | 03810-L01211 | | | 3p25 |
| ESR1 | estrogen receptor 1 | | 02202-L01700 | | | 6q25 |
| RASSF1 | Ras association (RalGDS/AF-6) domain family member 1 | | 03807-L02159 | | | 3p21 |
| TP73 | Tumor protein p73 | | 04050-L01263 | | | 1p36 |
| FHIT | fragile histidine triad | | 02201-L01699 | | | 3p14 |
| CADM1 | cell adhesion molecule 1 | | 03819-L03848 | | | 11q23 |
| CDH13 | Cadherin 13, H-cadherin | | 07946-L07727 | | | 16q23 |
| GSTP1 | glutathione S-transferase pi 1 | | 01638-L01176 | | | 11q13 |

Supplemental Data 2 Genes in the methylation-specific MS-MLPA kit (ME001-C1) Tumor suppressor

| Gene | Primer | | Size(bp) |
| --- | --- | --- | --- |
| CASP8  NM_001080125.1 | Forward | 5’- AGGGGTTATTATTATTAAATGGAGTTAGTA-3’ | 63 |
|  | Biotinylated-reverse | 5’- AATACCCAATTTCCAACCATTCAA-3’ |  |
|  | Sequencing primer | 5’- ATGTTTTTTAATAAAGTATGTTTAG-3’ |  |
| FHIT  NM_002012.2 | Forward | 5’- GGGAGGTAAGTTTAAGTGGAATATTGT-3’ | 47 |
|  | Biotinylated-reverse | 5’- AATCCCCACCCTAAAACCCTC -3’ |  |
|  | Sequencing primer | 5’-GGTTATTATTTAGGAGTTTAGTGG-3’ |  |
| CHFR  NM_001161344.1 | Forward | 5’- AGATTAGAGGGGTTTTTAGAATTTT-3’ | 36 |
|  | Biotinylated-reverse | 5’- ACCATCTTTAATCCTAACCAAAC -3’ |  |
|  | Sequencing primer | 5’- GTTTTTTTTGTTTTAATATAATATG-3’ |  |

Supplemental Data 3 The primers for Pyrosequencing

| Gene | Primer | | Size(bp) |
| --- | --- | --- | --- |
| *CASP8* | Forward primer | 5’- AAGTGAGCAGATCAGAATTGAGG-3’ | 72 |
|  | Reverse primer | 5’- CATCCAGTTTGCATTTGGAG-3’ |  |
| *FHIT* | Forward primer | 5’- TTTGGCCAACATCTCATCAA-3’ | 90 |
|  | Reverse primer | 5’- TCCTGGTACCACAGGTTTCC-3’ |  |
| *CHFR* | Forward primer | 5’- TGTTCCATGGGACCAAAGAT-3’ | 168 |
|  | Reverse primer | 5’- GAGAAGGCTCCGTGGAAGAG-3’ |  |

Supplemental Data 4 The primers for Real-time PCR


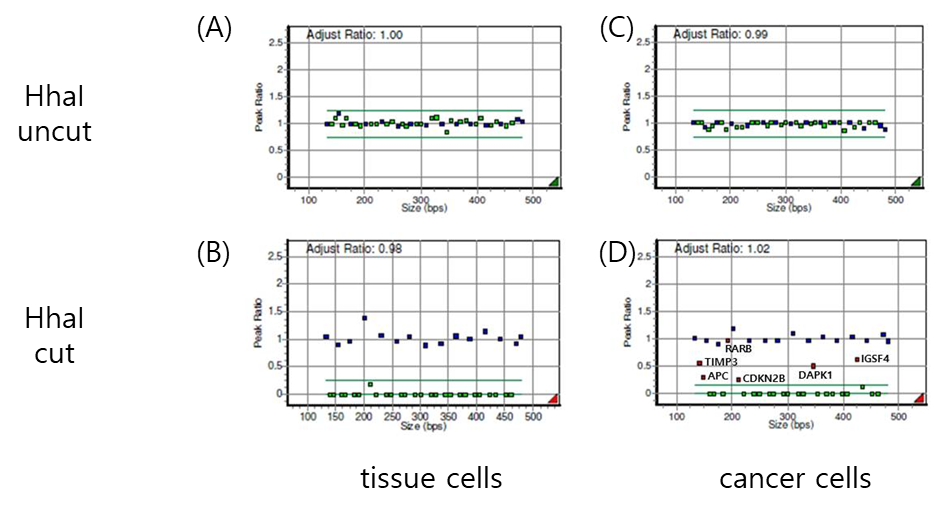


Supplemental Data 5

Methylation frequencies (cut-off value 25%) of the 25 promoter region in normal tissue cells and cancer tissue cells (A): In the absence of HhaI restriction enzyme treatment, tumor suppressor probes (green dots) and control probes (blue dots) were amplified without enzyme digestion in normal tissue cells. (B): Under HhaI restriction enzyme treatment, only the control probes (blue dots) were amplified in normal tissue cells. (C): In the absence of HhaI restriction enzyme treatment, tumor suppressor probes (green dots) and control probes (blue dots) were amplified without enzyme digestion in cancer cells. (D): Under HhaI restriction enzyme treatment, the control probes (blue dots) and some of tumor suppressor probes (red dots) were amplified in cancer cells.


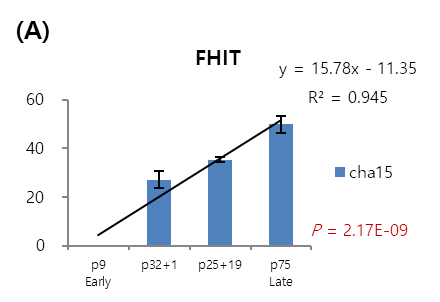

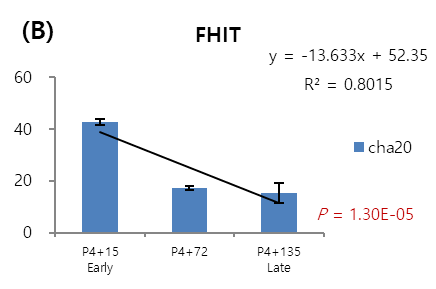


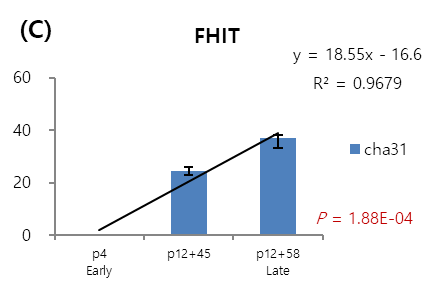

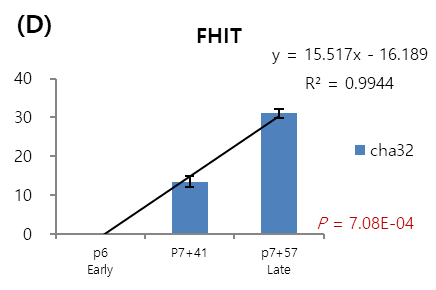


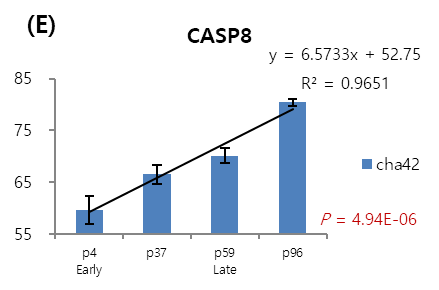

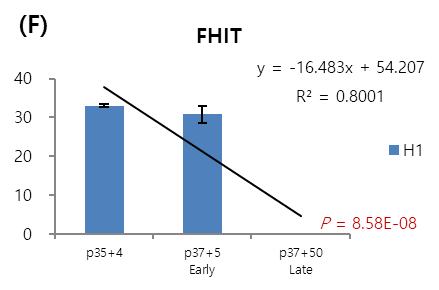


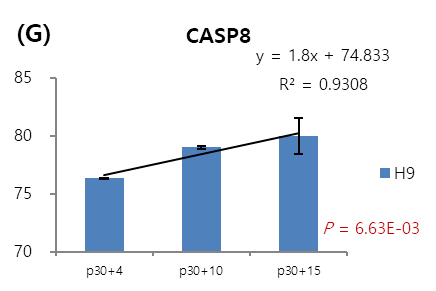

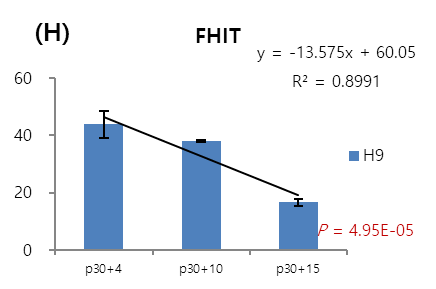


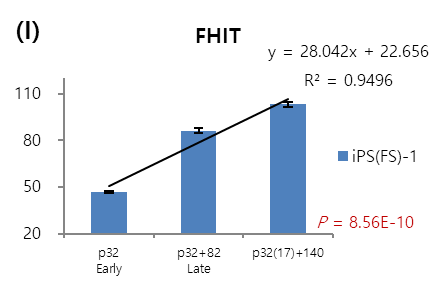

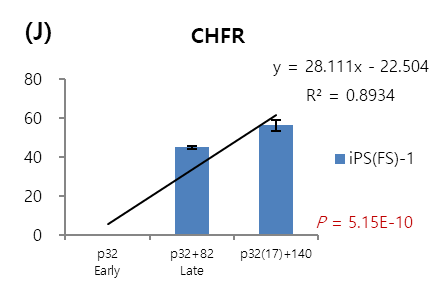


Supplemental Data 6

(A)~(J): MS-MLPA was performed by randomly taking middle passage cell lines between early passages and late passages, and it was confirmed that their methylation values were within the trend lines of early and late passages. ‘Early’ and ‘Late’ under passage means the cell line used previous experiment. In (F), (I) and (J), early passage or late passage cell lines were used as an exception because it was difficult to use middle passages.
